# Association Between Weather Parameters and SARS‐CoV‐2 Confirmed Cases in Two South African Cities

**DOI:** 10.1029/2021GH000520

**Published:** 2022-11-01

**Authors:** Samuel Ogunjo, Adeyemi Olusola, Israel Orimoloye

**Affiliations:** ^1^ Department of Physics Federal University of Technology Akure Nigeria; ^2^ Faculty of Environmental and Urban Change York University Toronto Canada; ^3^ Department of Geography University of the Free State Bloemfontein South Africa; ^4^ Department of Geography, Faculty of Food and Agriculture The University of the West Indies, St. Augustine Campus St. Augustine Trinidad and Tobago

**Keywords:** COVID‐19, Granger causality, Pretoria, Cape Town, lag

## Abstract

Several approaches have been used in the race against time to mitigate the spread and impact of COVID‐19. In this study, we investigated the role of temperature, relative humidity, and particulate matter in the spread of COVID‐19 cases within two densely populated cities of South Africa—Pretoria and Cape Town. The role of different levels of COVID‐19 restrictions in the air pollution levels, obtained from the Purple Air Network, of the two cities were also considered. Our results suggest that 26.73% and 43.66% reduction in PM2.5 levels were observed in Cape Town and Pretoria respectively for no lockdown (Level 0) to the strictest lockdown level (Level 5). Furthermore, our results showed a significant relationship between particulate matter and COVID‐19 in the two cities. Particulate matter was found to be a good predictor, based on the significance of causality test, of COVID‐19 cases in Pretoria with a lag of 7 days and more. This suggests that the effect of particulate matter on the number of cases can be felt after 7 days and beyond in Pretoria.

## Key Points

- Levels of particulate matter within two cities are modulated by COVID‐19 restrictions
- Empirical assessment of the interaction between COVID‐19 and particulate matter yields a complex relationship
- Local climate and human activities affect the influence of temperature, relative humidity, and particulate matter on COVID‐19 cases

## 1 Introduction

The natural environment has been grappling with major environmental problems, such as but not limited to climate change, biodiversity loss, ocean acidification, diminishing access to freshwater, resource waste and hazardous pollutants (Miller & Spoolman, 2011; Real, 1996), amplified as a result of various anthropogenic activities. However, added to this list is the emergence and re‐emergence of infectious diseases in recent times. Together, these problems are a result of the continued unsustainable ways through which humans interface with the natural environment (Real, 1996). The persistence in the use of earth's natural resources without regard to the principles of sustainability to a large extent opens up an avenue for chaos and disasters such as we have been experiencing in different spheres of the environment. The emergence and re‐emergence of infectious diseases presuppose that there is the need to move away from the traditional analyses of ecological and public health systems to a more integrated approach that is holistic in nature such as the One World One Health (OWOH) (Hinchliffe, 2015; Zinsstag et al., 2011).

OWOH is an approach that looks at the “critical zone”, in this case, the human, animal and environmental interface (Zinsstag et al., 2011). It has gained so much acceptance and has ascended in prominence as a result of the emergence and re‐emergence of infectious diseases in recent times because the majority of these diseases are zoonotic (Hinchliffe, 2015; Zinsstag et al., 2011). Examples of these diseases are but are not limited to Zoonotic influenza, Salmonellosis, West Nile Virus, Plague, Emerging Coronaviruses, Rabies, Brucellosis, and Lyme Disease (Awogbindin et al., 2021; Mayer, 2000; A. Olusola et al., 2020; Ayolabi et al., 2019; Onafeso et al., 2021; Campbell et al., 2002; Wu et al., 2016; Bourhy et al., 1999; LoGiudice et al., 2003; Bonilla‐Aldana et al., 2020; Motayo et al., 2019). As indicated by the World Health Organization, the conditions creating the emergence of new and re‐emergence of old diseases are likely to worsen in the future (Real, 1996) due to activities such as but not limited to land use changes, lifestyle and nutrition especially in some parts of the world. Largely interwoven with this especially in Sub‐Saharan Africa and in some other emerging and developing economies across the world is the issue of poverty, governance, and weak institutions (A. Olusola et al., 2020; Onafeso et al., 2021). It is sufficient to know that most of these developing economies are by and large the custodians of vast biodiversity that are becoming degraded and depleted as a result of increasing demand for natural capital accumulation by developed economies (Chamberlain, 2014; Michalopoulos & Papaioannou, 2016; Southall & Melber, 2009). These factors aid the persistent interactions in an unsustainable manner between humans, animals and their environments. These factors in no small means are predisposing the human populace to these diseases and creating a fragile world in terms of health and environment.

The impact of these diseases on man, animals and the environment is very grave. Overall, it reduces life expectancy, economic loss, biodiversity loss and establishes a new‐normal in the natural environment. These diseases asides from the fact that they affect humans in no small means, also affect agricultural production as seen in the case of Sugarcane Rust and White Pine Rust in the United States of America, Ebola in East and West Africa, etc. The case of the Sugarcane Rust and White Pine Rust in the United States of America affected cultivars in no small measures and the spread was alarming. The impact on production and extraction of juice was badly impacted. Added to these issues are the ongoing global concern as regards climate change and natural capital depletion. At present, the resilience of the human fabric is being challenged on every front. Global and regional pandemics prevent the achievements of Sustainable Development Goals such as zero hunger, sustainable human settlements, no poverty, good health and well‐being, and clean water and sanitation. Every society is working to achieve these sustainable development goals with the available resources in their domain. Already, every national, regional and provincial governments are carrying a heavy load as to how to achieve the sustainable goals before the emergence of COVID‐19. The emergence of the Coronavirus in December 2019, in Wuhan City, Hubei Province, China (A. Olusola et al., 2020; Onafeso et al., 2021) not only put a strain on the already fragile human‐ecosystem fabric but also suggests that the ongoing and constant interactions between man and his environment should be revisited. These interactions create varied land‐use patterns that invariably provide host structure for virus mutation such as COVID‐19 (A. Olusola et al., 2020; Syal, 2021; Snedden et al., 2021; Lloyd‐Smith, 2013). The ongoing pattern, mutation and distribution of the COVID‐19 has renewed interest in the factors that help perpetuates the survival of the host and the replica of the pathogen itself. As against the traditional idea of host‐pathogen assuming a benign state, COVID‐19 and its mutations such as the Delta Strain (Roy et al., 2021) and others have shown that if the replication rate of a pathogen is associated with the effects on the host (virulence) then strains with high replica rates (high virulence) are likely to replace strains of low virulence due to the straightforward advantage of an increased replica (Real, 1996). For example, variants such as Omicron is likely to replace other variants of interests or concern because it has a very high transmission rate which is likely due to its immune escape capabilities and high replicating rates (Papanikolaou et al., 2022; Tiecco et al., 2022). The question then is what are the likely factors aiding the transmission of COVID‐19 in the environment and what is the human‐environment‐COVID‐19 relationship like? There are reasons to believe that as a result of changing land use patterns and other anthropogenic activities leading to increasing aerosols and variability in weather parameters, COVID‐19 morbidity could be enhanced (B. Wang et al., 2020; Yao et al., 2020; Contini & Costabile, 2020; B. Chen et al., 2021; Srivastava, 2021; Jayaweera et al., 2020; Küpper et al., 2020; Sosnowski, 2021).

Association between particulate matter, population mobility and COVID‐19 has been established in several regions of the world. Some of these studies claim that low temperature, mild diurnal temperature range, and low humidity likely contribute to the transmission of the disease. Furthermore, it has been established that virus‐accumulating aerosols are easily transmitted among individuals (B. Wang et al., 2020; Yao et al., 2020; Contini & Costabile, 2020; Tellier et al., 2019; Tang et al., 2006; Liu et al., 2020; Wu, Jing et al., 2020; Sehra et al., 2020). These aerosols are likely composed of airborne pollution particles and attached virus droplets, which promote the spread of pathogens such as influenza viruses. Several studies (Atkinson et al., 2014; R. Chen et al., 2017; Yao et al., 2020) posited that exposure to ambient particulate matter is likely to increase the risks of mortality and morbidity of cardiopulmonary diseases worldwide with the elderly and those with underlying medical conditions likely to be within the fatal brackets from COVID‐19 (Guan et al., 2020). Furthermore, some recent studies have been found reporting the relationship between COVID‐19 and meteorological variables (temperature, relative humidity, particulate matter), taking into account the climatic variability that exists in different parts of the world and addressing the question of whether climatic factors have influenced the spread of the SARS‐Cov‐2 virus (Ahmadi et al., 2020; Biktasheva, 2020). For instance, Ahmadi et al. (2020) investigated the effects of climatic parameters on the COVID‐19 outbreak in Iran and the study found that temperature and precipitation have a positive relationship with the number of infected cases, whereas humidity, wind speed, and solar radiation have a negative relationship. Biktasheva (2020) discovered that in German federal states, local air humidity had a negative connection with COVID‐19 mortality. Absolute humidity has a positive link with death tolls, according to Ma et al. (2020). In contrast, Qi et al. (2020) concluded that both temperature and humidity are adversely connected with COVID‐19 transmission in Mainland China, and Sahin (2020) found almost similar results using meteorological factors for major Turkish cities. Pal and Masum (2021) investigated the relationship between COVID‐19 cases and meteorological parameters to predict COVID‐19 transmission over a long period and across different climatic patterns. They discovered that there were significant positive associations between relative humidity and COVID‐19 cases across cities, while temperature had both positive and negative associations. Prediction of COVID‐19 cases within Nigeria using machine learning algorithms have been carried out (Ogunjo, Fuwape et al., 2022). The association between particulate matter and COVID‐19 cases has been established for 17 out of 44 United States cities using the Granger causality test (Kamigauti et al., 2021). In a study of 20 countries, meteorological factors have been adjudged to be good predictors of COVID‐19 cases based on the Granger causality test (Mirri et al., 2021; Sarkodie & Owusu, 2020). Atmospheric parameters such as temperature and humidity have been found to play important role in the transmission of COVID‐19 within Nigeria (Ogunjo, Olaniyan et al., 2022). It is clear from previous and relevant studies that weather parameters may have associations with COVID‐19 cases and that variation in transmission and death tolls are not uncommon in different regions; thus, a few more local/regional studies may not only help in consolidating the impact of climatic patterns from the less reported countries and localities, for instance, South Africa, but also serve as a useful stepping stone to further research though not without its challenges.

One of the key challenges is the fact that ascertaining the mode of transmission as per COVID‐19 is nearly impossible. Contact tracing and all other means are largely inconclusive but could provide an idea (Kleinman & Merkel, 2020; Mbunge, 2020). As stated by World Health Organization, contact tracing is a key component of a public health response to infectious disease outbreaks. Contact tracing is a key strategy for interrupting chains of transmission of SARS‐CoV‐2 and reducing COVID‐19‐associated mortality (Braithwaite et al., 2020; Kleinman & Merkel, 2020; Mbunge, 2020). One of the take home from contact tracing during COVID‐19 has been that the transmission is through coming in contact with an infected person or “fomite” (i.e., an object or surface that has been contaminated with the virus) (Meiksin, 2020; Wilson et al., 2021; T. Chen, 2021). Transmission through the air or airborne transmission is believed to not require direct contact. These could be through aerosols that are virus‐laden or droplets that are virus‐laden. As stated by World Health Organization, airborne transmission is different from droplet transmission as it refers to the presence of microbes within droplet nuclei, which are generally considered to be particles <5 μm in diameter, can remain in the air for long periods and be transmitted to others over distances greater than 1 m.

In this study, we aim to investigate the impact of COVID‐19 associated lockdown on air quality in two densely populated cities of South Africa. To achieve the aim of this study, we will highlight the contributions of particulate matter and atmospheric parameters on the number of COVID‐19 cases within two cities in South Africa. In addition, the study explores the probability trend of COVID‐19 transmission using statistical tools to portray the association of COVID‐19 cases with meteorological variables, which can be helpful to check and plan for future occurrences. Few to no study has been done over South Africa, except a study by Meo et al. (2020) which investigated the impact of weather conditions, heat and humidity on the daily incidence and mortality due to COVID‐19 pandemic in 16 highly populated African countries, including South Africa, Ghana, Nigeria, Egypt, Algeria, Morocco, Kenya, Congo, Ivory Coast, Cameroon, Niger, Somalia, Tanzania, Zambia, Mozambique, Madagascar. This study focuses on the need to appraise the relationship between meteorological parameters and COVID‐19 in South Africa. As a result, it would be worthwhile to explore the relationship between meteorological characteristics and COVID‐19 cases to identify likely scenarios. The association between meteorological parameters (temperature, relative humidity, and particulate matters) and the ongoing pandemic pattern (COVID‐19) has not yet been adequately examined in South Africa, to our knowledge.

## 2 Methodology

### 2.1 Study Area and Data

**Figure 1:**
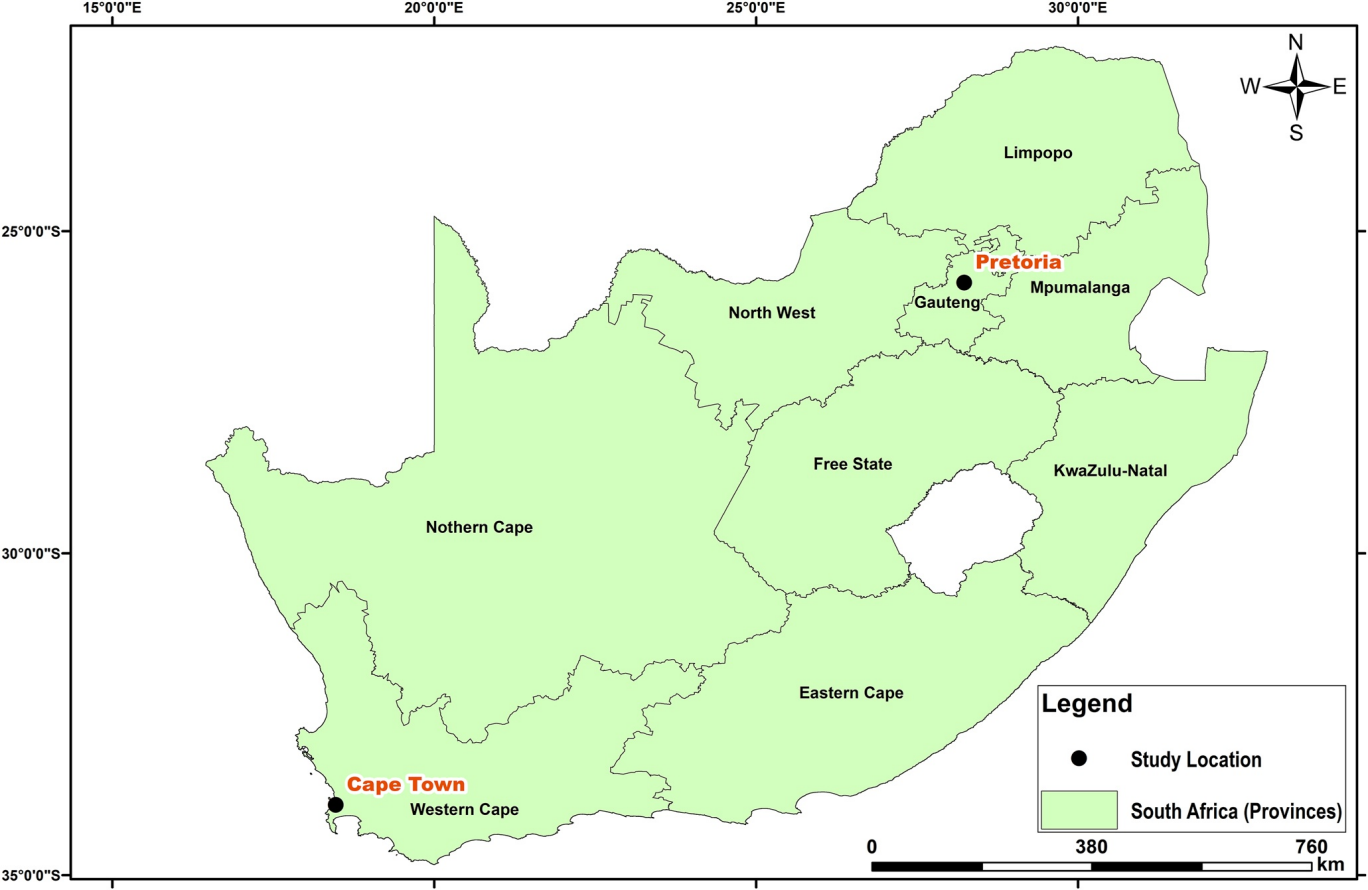
Map of South Africa showing Pretoria and Cape Town source: Google Earth Engine.

**Figure 2:**
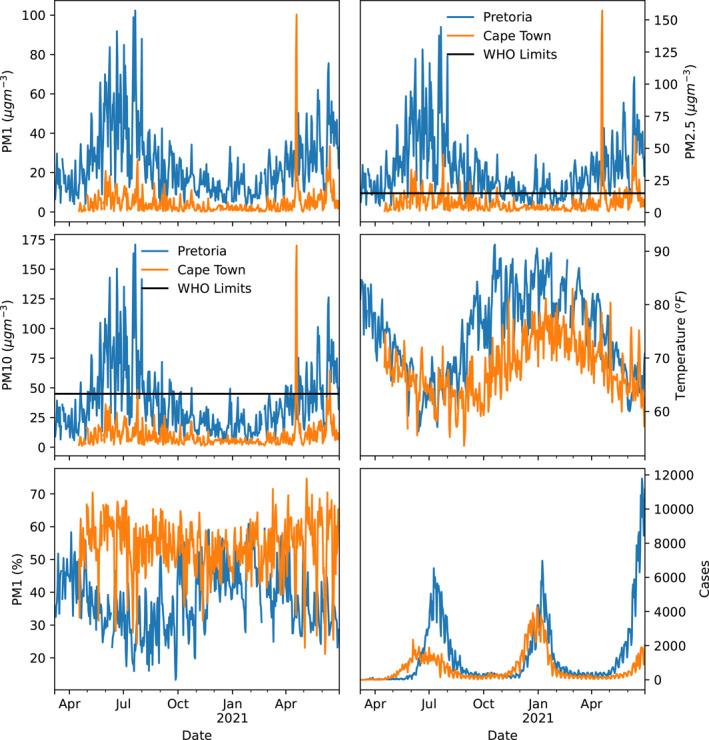
Temporal variation of PM1, PM2.5, PM10, temperature, relative humidity, and daily COVID‐19 cases at Pretoria and Cape Town during the period under investigation.

Two populated cities in South Africa were considered in this study, that is, Cape Town and Pretoria (Figure 1). Cape Town, a port city in South Africa and the seat of the parliament, is located at latitude 33*°*55′31”S and longitude 18*°*25′26”E. Cape Town being the oldest urban center in the Western Cape. The Cape Colony's commercial and cultural hub, Cape Town, surpassed its intended function as the first European foothold at the Castle of Good Hope. It has a population of about 1,619,000 (https://www.statista.com/statistics/1127496/largest‐cities‐in‐south‐africa/). Cape Town was the largest city in South Africa until the Witwatersrand Gold Rush and the establishment of Johannesburg. The climate in Cape Town is Mediterranean, with mild, moderately rainy winters and dry, pleasant summers (Schumann & Martin, 1991). Large cold fronts from the Atlantic Ocean, with heavy precipitation and strong north‐westerly winds, may penetrate for brief times throughout the winter, which runs from the beginning of June until the end of August. The city's average winter temperature is 18*°*C (64*°*F) with a minimum of 8.5*°*C (47*°*F). The city's annual rainfall is 515 mm (20.3 in), while rainfall in the Southern Suburbs, adjacent to the mountains, is much greater, averaging closer to 1,000 mm (39.4 in). Summer, which lasts from December to March, is hot and dry, with average highs of 26 degrees Celsius (79 degrees Fahrenheit) and lows of 16 degrees Celsius (61 degrees Fahrenheit) (Scott et al., 2019). Pretoria, on the other hand, is located farther inland, northeast of Johannesburg and serves as a transitional belt. It is located at latitude 25*°*44′46”S and longitude 28*°*11′17”E. Pretoria is one of South Africa's three capital cities, serving as the seat of the executive branch of government and the home of the country's foreign embassies. With the Tshwane University of Technology, the University of Pretoria, the University of South Africa, the Council for Scientific and Industrial Research, and the Human Sciences Research Council, Pretoria has a reputation as an academic city and a research hub. The National Research Foundation and the South African Bureau of Standards are also housed in the city. Pretoria's climate is humid subtropical, with long, hot, rainy summers and short, moderate winters. Winters in the city are typical of South African winters, with chilly, clear nights and mild to moderately warm days (Conradie, 2018; Kumar et al., 2020). Although the typical low temperatures throughout the winter are mild, the clear skies can make it feel chilly, with nighttime low temperatures in previous years ranging from 2 to 5°C (36–23°F). Pretoria's population ranges from 700,000 to 2.95 million (https://www.statista.com/statistics/1127496/largest‐cities‐in‐south‐africa/). Summarily, South Africa is a subtropical country, moderated as a result of the oceans and altitude of the interior plateau. The country is relatively dry with an average annual rainfall of about 460 mm. The Cape areas receive most of their rainfall in winter while the rest of the country receives theirs during the summer. Due to its elevation above sea level, South Africa has lower temperatures compared to other countries at similar latitudes. As a result of the elevation gradient, interior locations such as the Pretoria keeps average summer temperatures, while the coastal regions such as the Cape are relatively warm in winter (www.gov.za). Data at the two locations were acquired from 7 March 2020 till 30 June 2021. There was a total of 6 and 43 missing data points, which represent less than 1% of the total, in Pretoria and Cape Town respectively. The temporal evolution of the parameters considered are shown in Figure 2.

The paucity of air quality monitoring facilities has been highlighted within Sub‐Saharan Africa (Amegah, 2018), East Africa (Singh et al., 2021) and South Africa (Naidoo et al., 2006). Over the years, different campaigns have been launched for air quality monitoring within the continent. These campaigns include: Dynamics–Aerosol–Chemistry–Cloud Interactions in West Africa (Deroubaix et al., 2019), ObseRvations of Aerosols above CLouds and their intEractionS (Ferrada et al., 2018), South African Air Quality Information System (Gwaze & Mashele, 2018), and Geostationary Earth Radiation Budget Intercomparison of Longwave and Shortwave radiation (Yang et al., 2009). However, most of these campaigns are short‐term and expensive. The limited air pollution measurements within the continent has led to the use of satellite and reanalysis data for COVID‐19 studies (I. Fuwape et al., 2021). In recent times, there have been several deployments of low‐cost sensors for air quality monitoring on the continent. Some of the low‐cost sensors deployed are Purple Air and Clarity Air sensors. In several studies, PurpleAir sensors have been found to have good performance with standardized equipment (Ardon‐Dryer et al., 2020; Bi et al., 2020; Li et al., 2020; Magi et al., 2020). In this study, Purple Air sensors (www.purpleair.com) located in Cape Town and Pretoria were considered. The sensors, one in each location were installed in 2019, reports PM1, PM2.5, and PM10, temperature and humidity data at 10 min interval. The 10 min data were aggregated to obtain the daily averages. Data for daily COVID‐19 cases were retrieved from SA COVID‐19 Interactive Dashboard (https://www.covid19sa.org/provincial‐breakdown).

### 2.2 Methods

#### 2.2.1 Linear and Quantile Regression

Linear regression provides a quantitative relationship about the response of a dependent variable (*y*) to an independent quantity (*x*). Regression methods are simple and cost‐effective approach for investigating the impact of meteorological impact on disease transmission (Wu, Nethery et al., 2020). This is expressed mathematically as

(1) $$y=\beta_{0}+\beta_{1}x+\epsilon_{i}$$

where beta_0_ and beta_1_ are referred to as the intercept and slope respectively. *ϵ*_*i*_ is the error term. The slope indicates the rate of change in COVID‐19 cases with respect to a unit of the predictor. Conditions for linear regression include: normality, linearity, independence, and homoscedasticity. In situations where these conditions are not met, quantile regression is often considered. The quantile regression at *p*th quantile is defined as

(2) $$Q_{y|x}\left(p\right)=\beta x$$

Since various quantiles of the data are considered, quantile regressions allowed for investigation of relationship over the whole data rather than around the mean (Bashir et al., 2021). Several studies related to COVID‐19 have been conducted using linear regression (Dettori et al., 2021; Rouen et al., 2020) and quantile regression (Azimli, 2020; Bashir et al., 2021; Irfan et al., 2022; Razzaq et al., 2020; Sarwar et al., 2021).

#### 2.2.2 Granger Causality Test

The Granger causality test Granger (1969) was employed to determine if the daily number of cases in each city can be predicted based on the considered atmospheric and particulate matter parameters. The independent variable *x*, is said to Granger cause the dependent variable *y*, if there exists a suitable lag, *q* such that (Granger, 1969; I. A. Fuwape et al., 2016)

(3) $$y_{t}=\beta_{0}+\sum_{j=1}^{q}c_{j}x_{t-j}+\sum_{k=1}^{q}d_{k}y_{t-k}$$

*F*‐test was used to test the validity of the null hypothesis. Each of the parameters were tested for stationarity using the Kwiatkowski–Phillips–Schmidt–Shin (KPSS) test before analysis for Granger causality. All parameters considered in Cape Town became stationary after first order differencing except temperature which required a third order differencing. Similarly, all parameters in Pretoria require first order differencing for stationarity except the number of cases which was stationary. This approach has been used to investigate the relationship between COVID‐19 cases and fatalities with various atmospheric parameters (Becchetti et al., 2020; Habib et al., 2021; Kamigauti et al., 2021; Pata, 2020; Tauqir & Kashif, 2021).

## 3 Results and Discussion

The statistical summary of parameters considered in this work is presented in Table 2. Mean values of all parameters for the period under review were observed to be higher in Pretoria compared to Cape Town, except for relative humidity. The high relative humidity and low temperature recorded in Cape Town can be attributed to the influence of the ocean. The daily mean values of PM2.5 and PM10 reported for Cape Town were far lower than the World Health Organization's recommended value of 15μgm^−3^ and 45μgm^−3^ with no more than 3–4 days exceedance days per year while the PM2.5 values obtained at Pretoria exceeded the recommended limits (World Health Organization, 2021). The minimum values in particulate matter in both locations were below the recommended limits, however, the maximum values exceeded the limits in both locations. The distribution of particulate matter are positively skewed, using the normal distribution, in both Cape Town and Pretoria but the values in Cape Town are more positively skewed than the values in Pretoria. The skewness in temperature and humidity present an interesting scenario at the two locations. Temperature was found to be negatively skewed in Pretoria but positively skewed in Cape Town. This implies that temperature data contains more high values than low values in Pretoria while temperature in Cape town has more low values than high values. The skewness in temperature is related to the closeness of each location to the ocean. Cold winds from the ocean tend to cool Cape Town, resulting in frequent low temperatures compared to Pretoria which is an inland station. The converse was obtained for relative humidity—it was negatively skewed in Cape Town but positively skewed in Pretoria. This means that relative humidity is dominated by high values in Cape Town but low values in Pretoria. Cape Town and Pretoria have COVID‐19 cases which vary from 0 to 4,241 and 0 to 11,782 per day respectively. The value obtained for Kurtosis in the two locations indicate higher outliers in particulate matter in Cape Town compared to Pretoria. The values of Kurtosis for particulate matter in the two locations were found to be heavy or fat‐tailed (Leptokurtic). Temperature and relative humidity in the two locations does not show a significant presence of outliers due to the low values of kurtosis obtained. However, a number of COVID‐19 cases were found to be heavy tail (Kurtosis greater than 3) in both locations. The outliers are in the COVID‐19 cases are due to the different waves within the study period.

**Figure 3:**
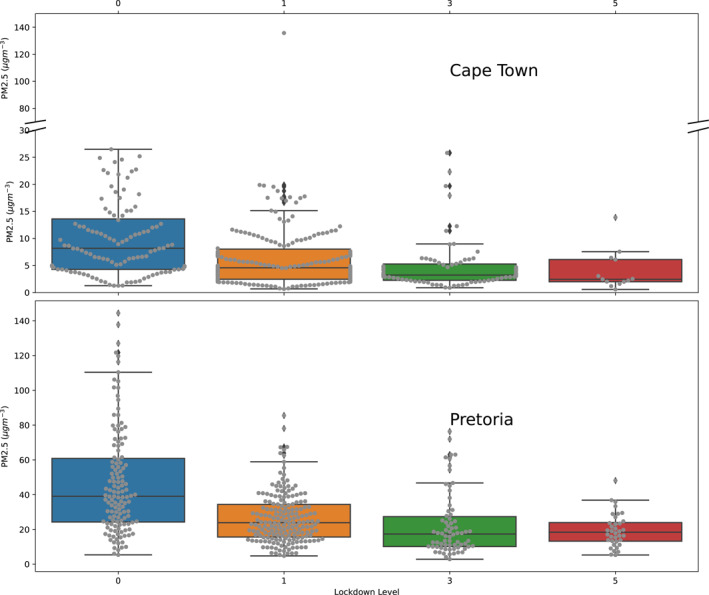
Statistical distribution of PM2.5 at various lockdown levels for Cape Town (top plot) and Pretoria (bottom plot). Colored boxes represent the third and first quantiles while the middle line in the boxes are the mean values. The gray lines represent the data points.

The variation of PM2.5 quantity to different levels of lockdown in Table 1 from no lockdown (Level 0) to the strictest lockdown level (Level 5) were considered (Figure 3). The values obtained were generally higher in Pretoria compared to Cape Town. In the absences of human movement restriction (Lockdown level 0), PM2.5 was found to have wider variations in both locations. Pollution in Cape Town has been influenced by four air masses from the Atlantic and Indian oceans (Williams et al., 2021). However, the pollution levels in Pretoria have been attributed to anthropogenic sources such as biomass burning, industrial pollution, and vehicular emission (Muyemeki et al., 2021). Imposing a Level 1 lockdown causes a 27.75%, 26.73%, and 25.34% reduction in PM1, PM2.5, and PM10 respectively with respect to the mean values at Cape Town relative to the no‐lockdown scenario while a reduction of 42.23%, 43.66%, and 45.42% was observed in PM1, PM2.5, and PM10 respectively at Pretoria. The reduction in PM1, PM2.5, and PM10 were much higher during a Level 3 lockdown in Cape Town with 58.31%, 54.48%, and 50.84%. The reduction was much lower at Pretoria with 51.32%, 51.78%, and 52.73% for a similar lockdown level. The greatest reduction due to COVID‐19 restrictions was reported at Lockdown Level 5 in both cities studied. In Cape Town, 67.02%, 61.94%, and 58.30% reduction was observed in PM1, PM2.5, and PM10 respectively, however, 58.16%, 58.42%, and 60.41% reduction were reported in Pretoria for PM1, PM2.5, and PM10 respectively. In Cape Town, higher percentage reductions were noticed in PM1 compared to PM10. This contrasts with the trend in Pretoria where the greatest reduction was in PM10 compared to PM1. The reductions in particulate matter levels in the two cities are within the range (33%) reported for Johannesburg for PM2.5 and PM10 (Fu et al., 2020) and 40.8% reduction in PM2.5 in Uganda (Rodríguez‐Urrego & Rodríguez‐Urrego, 2020). These values were higher than the 35% reduction reported for Uganda and the 40% reduction in Kinshasa (McFarlane et al., 2021).

**Table 1:** Details of Different Lockdown Levels Within South Africa

Lockdown levels	Description	Time‐period
Level 5	Drastic measures are required to contain the spread of the virus to save lives.	26 March to 30 April 2020;
Level 4	Some activity can be allowed to resume subject to extreme precautions required to limit community transmission and outbreaks.	1 to 31 May 2020; from 16 June 2021; 8 June to 25 July 2021
Level 3	Involves the easing of some restrictions, including on work and social activities, to address a high risk of transmission.	1 June to 17 August 2020; 29 December 2020 to 28 February 2021; 16 June to 27 June 2021; 26 July to 12 September 2021
Level 2	Further easing of restrictions, but the maintenance of physical distancing and restrictions on some leisure and social activities to prevent a resurgence of the virus.	18 August 2020; 31 May to 15 June 2021; from 13 September 2021
Level 1	Most normal activity can resume, with precautions and health guidelines followed at all times.	21 September to 28 December 2020; 1 March to 30 May 2021; 1 October 2021

*Note*. Source: https://www.gov.za/covid‐19/about/about‐alert‐system.

**Table 2:** Statistics of Particulate Matter, Atmospheric Parameters, and Mean COVID‐19 Cases at Cape Town and Pretoria From 7 March 2020 Till 30 June 2021

Statistics	Location^a^	PM1	PM2.5	PM10	Temperature	Humidity	Cases
Mean	Cape Town	4.37	7.89	9.45	68.61	53.18	668.22
	Pretoria	22.43	30.02	34.21	76.40	38.50	1,344.71
STD	Cape Town	7.23	11.68	12.55	5.69	8.91	826.66
	Pretoria	16.62	22.72	27.14	8.32	9.20	2,018.75
Min	Cape Town	0.01	0.16	0.39	53.55	21.19	0.00
	Pretoria	2.25	2.85	3.13	56.24	13.32	0.00
Max	Cape Town	100.30	157.25	169.97	83.00	74.66	4,241
	Pretoria	102.34	144.42	170.82	91.58	62.79	11,782
Skew	Cape Town	8.15	7.88	7.76	0.11	−0.78	1.95
	Pretoria	1.82	1.86	1.93	−0.29	0.06	2.43
Kurtosis	Cape Town	94.30	88.19	86.56	−0.59	0.91	3.60
	Pretoria	4.06	4.23	4.60	−0.88	−0.25	6.59

^a^ Particulate matters, temperature, and humidity are in units of μgm^−3^,°F, and % respectively.

Beyond Africa, a study carried out in Colombia, Arregocés et al. (2021) posited that lockdown levels as adopted in the country had a significant impact on the country's air quality. In addition, in another study carried out in Lahore, Pakistan, it was observed that as a result of the lockdown effect, even though the country recorded increasing COVID‐19 cases, the air pollution in the city reduces (Tauqir & Kashif, 2021). In another study conducted in India, the authors (Agarwal et al., 2021) observed that during the period of their study, vehicular movement was minimum as a result of COVID‐19 lockdown. In this study, they realised that the level of air pollutants; PM10, PM2.5, SO2 and NO2 were found to be 54, 41, 19, and 14 μg/m^3^, respectively which is minimum in the six months of study (January 2020–June 2020) and lowest in comparison to the air quality data of the last 25 years. The authors (Agarwal et al., 2021) came to the conclusion that as a result of lockdown which invariably reduces vehicular movement, there was a significant improvement in air quality across Bareilly in Uttar Pradesh, India. Furthermore, as observed in this study and many others, the role of COVID‐19 lockdown across various countries shows that anthropogenic and other factors, if they can be reduced, would greatly help in maintaining good air quality especially in urban centers (Rita et al., 2021). Rita et al. (2021) in their study posited that as a result of various lockdown levels engendered by the COVID‐19 pandemic, there was a positive impact on the environment due to a drastic reduction in greenhouse gasses emissions and other pollutants below pre‐COVID‐19 levels. In their study on the impact of COVID‐19 pandemic on nitrogen dioxide levels in Nigeria, J. A. Olusola et al. (2021) concluded that there was a significant reduction in NO2 levels during the lockdown period compared with its levels during the pre‐lockdown period in 2019. The reduction in NO2 according to the authors is likely due to less traffic, social distancing and restrictions on business and human activities. Even though there is a need for caution in the interpretation and acceptance of cause‐effect relationship between PM and COVID‐19 cases, several studies (Arregocés et al., 2021; Bianconi et al., 2020; Tung et al., 2021; Yao et al., 2020) have shown empirically the potential relationship between particulate matter and COVID‐19 cases as revealed in this study. In their study, Bianconi et al. (2020) highlighted the possible reasons behind PM‐actions and increasing vulnerability to COVID‐19. They listed reduced immune deficiencies, exacerbation of pre‐existing disorders, formation of PM‐viral complexes facilitating viral spread. They claimed that although the above listed are quite fascinating but still requires further research. In their study, Bianconi et al. (2020) concluded that exposure to PM especially 2.5 and 10 is linked with COVID‐19 deaths positing that PM might have a role to play in the outbreak and possible deaths as observed in some provinces in Italy. Yao et al. (2020) reported that the progression of COVID‐19 cases within 49 Chinese cities have a possible association with particulate matter exposure. They concluded that there is a valid case to believe that exposure to long term PM could be a determinant for increasing vulnerability to COVID‐19, a position supported by Tung et al. (2021).

**Figure 4:**
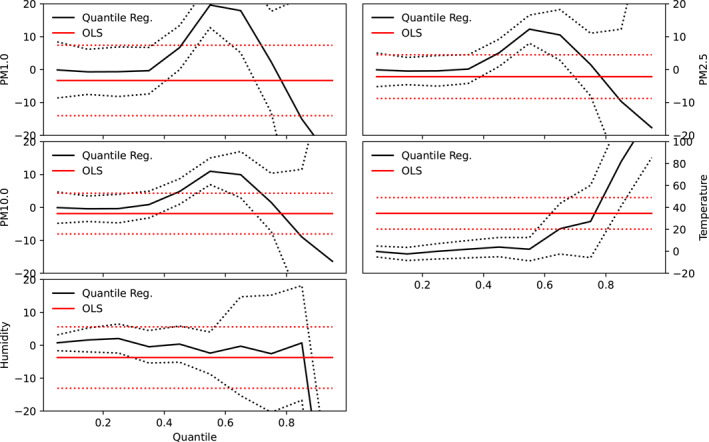
Co‐efficient of quantile regression and linear regression at Cape Town. Red and black solid lines represent the linear and quantile regression respectively while the red and black dotted lines indicate the 95% confidence interval.

**Figure 5:**
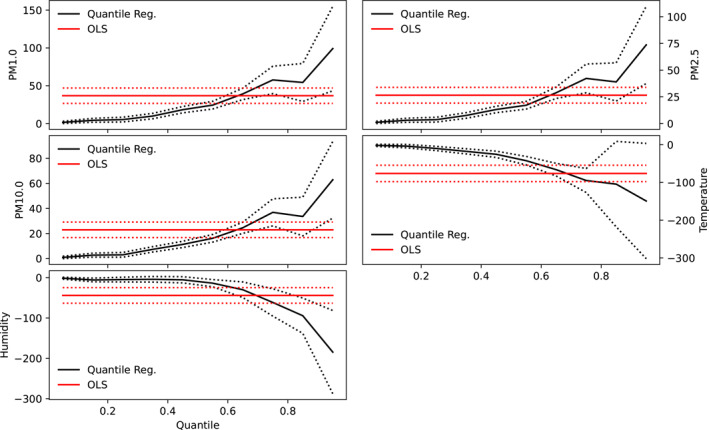
Co‐efficient of quantile regression and linear regression at Pretoria. Red and black solid lines represent the linear and quantile regression respectively while the red and black dotted lines indicate the 95% confidence interval.

The results of both quantile and linear regression analysis are shown in Figures 4 and 5 for Cape Town and Pretoria respectively. Parameters of both linear and quantile regression are shown in Table 3. Ordinary linear regression values of −3.28, −2.12, and −1.86 were recorded for PM1, PM2.5, and PM10 respectively in Cape Town, however, these values were not significant. This suggests that an inverse relationship exists between COVID‐19 cases and particulate matter in Cape Town. Compared with Pretoria, the linear regression model yields significant values of 36.81, 26.48, and 22.94 as slope values for PM1, PM2.5, and PM10 respectively. These suggest that particulate matter has significant relationship with the number of cases in Pretoria but not in Cape Town. It can be inferred that lower values of particulate matter correspond to lower case number in Pretoria while low values of particulate matter correspond to higher cases in Cape Town. Furthermore, a significant relationship was observed between temperature and the number of COVID‐19 cases in both cities, however, the relationship was negative in Pretoria but positive in Cape Town. Both cities exhibit a negative relationship between COVID‐19 cases and relative humidity, however, the relationship was only significant in Pretoria. The intercept of the linear regression were found to be significant at a 99% confidence interval in both cities for all predictors. Other studies reported slope values of COVID‐19 and PM2.5 regression line as −0.1895 (−0.4937) for PM2.5(PM10) in India (Selvi et al., 2021) and 1.1 × 10^−4^ for PM2.5 in Italian provinces (Borro et al., 2020). Negative relationship has been established between relative humidity and COVID‐19 cases in some studies (Ogunjo, Olaniyan et al., 2022; Wu, Nethery et al., 2020; J. Wang et al., 2021).

**Table 3:** Some Captions

Pretoria							
	OLS		Quantile regression				
Variable	Slope	Intercept	0.05	0.25	0.45	0.65	0.85
PM1	36.81 (5.18)***	456.53 (154.53)***	1.52 (0.77)**	5.21 (1.60)***	18.31 (2.14)***	39.19 (3.84)***	54.34 (12.77)***
PM2.5	26.49 (3.76)***	487.05 (151.9)***	1.05 (0.56)*	3.57 (1.15)***	13.11 (1.53)***	28.84 (2.74)***	38.95 (9.12)***
PM10	22.94 (3.15)***	493.95 (147.62)***	0.92 (0.50)*	2.99 (0.96)***	11.3 (1.28)***	24.58 (2.27)***	33.59 (7.85)***
Temperature Humidity	−76.13 (11.03)***	7,040.63 (828.45)***	−1.86 (1.46)	−10.04 (2.87)***	−25.45 (4.25)***	−66.23 (8.58)***	−104.51 (57.61)*
Cape Town	−44.09 (9.84)***	3,028.40 (383.71)***	−1.31 (1.25)	−4.76 (2.98)	5.33 (3.92)	−29.95 (9.92)***	−94.41 (22.29)***
PM1	−3.28 (5.44)	747.36 (47.34)***	−0.1 (4.33)	−0.64 (3.82)	6.69 (3.40)**	17.94 (6.53)***	−15.04 (18.74)
PM2.5	−2.12 (3.37)	749.86 (48.96)***	−0.06 (2.59)	−0.38 (2.36)	5.06 (2.10)**	10.54 (3.92)***	−9.65 (11.17)
PM10	−1.86 (3.15)	750.76 (50.89)***	−0.06 (2.42)	−0.35 (2.20)	4.84 (1.96)***	9.97 (3.58)***	−8.99 (10.47)
Temperature	34.48 (7.32)***	−1,614.00 (499.60)***	−0.23 (2.52)	−2.59e−8 (3.55)	3.82 (4.45)	0.21 (11.73)*	8.18 (20.61)***
Humidity	−3.75 (4.75)	934.91 (259.35)***	0.75 (1.22)	2.05 (2.24)	0.35 (2.79)	−0.28 (7.65)	0.71 (8.87)

*Note*. ***0.01, **0.05, *0.1.

At low quantiles, a small negative association was observed between the particulate matters and the number of COVID‐19 cases in Pretoria (Figure 4). The value ranges from 1.52 at 0.05 quantile to 54.34 at 0.85 quantiles for PM1. All the slope values were positive and statistically significant at all quantile levels. This suggests that at all quantiles, particulate matter significantly contributes to the number of COVID‐19 cases in Pretoria, however, the impact was found to be more at higher quantiles. It is inferred that large values of particulate matters contribute more to the number of cases compared to lower values. In a similar study carried out across Italian regions and provinces (Bianconi et al., 2020), the authors observed that exposures to PM2.5/PM10 were independently associated with COVID‐19 incidence proportion at the provincial level. They concluded in their study that particulate matter pollution may play a role in the COVID‐19 outbreak and explains the heterogeneous distribution of COVID‐19 in Italian regions and provinces. In another global study, on the global association between COVID‐19 cases and airborne particulate matter at the regional level, the authors (Solimini et al., 2021) concluded that there exists an association between COVID‐19 cases and air pollution suggestive of a possible causal link among particulate matter levels and incidence of COVID‐19. Conversely, the relationship between COVID‐19 and particulate matter in Cape Town were negative and non‐statistically insignificant at low and high quantiles but positive and statistically significant at mid‐quantiles (0.3–0.8). This suggests that extreme low and large values of particulate matter have significant impact on the observed number of cases in Cape Town. Temperature showed negative relationship, which is not significant at low quantile, with the number of cases in Pretoria. In Cape Town, the relationship between temperature and COVID‐19 cases was negative and not significant at low quantiles (between 0.05 and 0.25) but positive and significant at higher quantiles. In the same vein, relative humidity showed a negative relationship at low and high quantiles in Pretoria but largely positive relationship in Cape Town. The relationship was only significant at high quantiles in Pretoria but not significant at any quantile in Cape Town. Significant values at low quantiles suggests that low values of that parameter contribute to the number of cases while significant values at high quantiles imply that only large values contribute to the observed cases. Varying responses of COVID‐19 cases to atmospheric parameters using quantile regression have been reported for different cities of the world (Razzaq et al., 2020; Sarwar et al., 2021; Shahzad et al., 2020).

**Figure 6:**
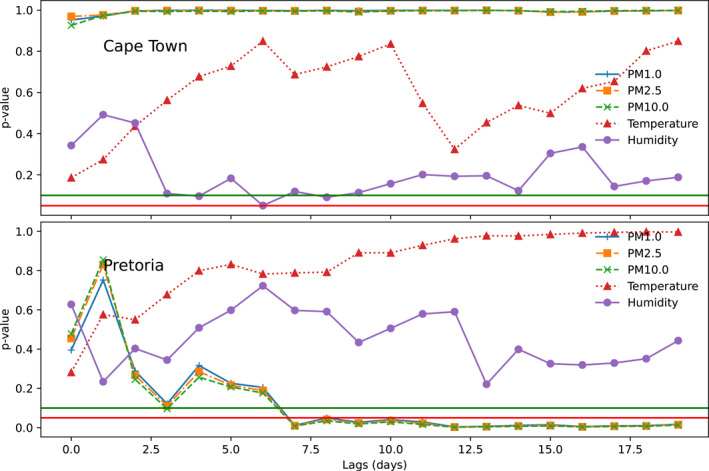
Statistical significance for the association between COVID19 cases and selected atmospheric parameters and particulate matter at 95% (red line) and 90% (green line) confidence interval.

Granger causality test was employed to estimate the lagged (0–21 days) effects of particulate matter and atmospheric parameters on COVID‐19 cases (Figure 6). Using this approach, the number of days before the effect of particulate matter and atmospheric parameters (temperature and relative humidity) reflect in the observed number of cases will be determined. Results obtained suggest that relative humidity has a casual link with COVID‐19 cases in Cape Town at 4 day lags (90% confidence interval) and 6 day lag (95% confidence interval). This implies that the impact of significant relative humidity events will have an impact on the number of cases within 4 or 6 days. These days were observed to fall within the incubation period of the virus. In Pretoria, the significance of temperature and relative humidity were above the 90% and 95% confidence interval. Hence, there is no evidence to suggest a causal relationship between temperature and humidity, and COVID‐19 cases in Pretoria. The causal relationships observed between COVID‐19 cases and particulate matter (PM1, PM2.5, PM10) at Pretoria is due to the significance of the Granger causality test observed for all the particulate matter after day 7. This suggests that the effect of particulate matter on the number of cases can be felt after 7 days and beyond in Pretoria. However, the causal relationship between particulate matter and the number of cases were not significant at both 90% and 95% confidence interval. It was observed that temperature has no casual relationship with COVID‐19 cases in both cities (Becchetti et al., 2020). Found a lag of 6–8 days between the incursion of particulate matter and COVID‐19 cases. The predictive capability of COVID‐19 from relative humidity can be attributed to the influence of ocean winds on the coastal city. The high relative humidity has also been suggested as responsible for daily all‐cause non‐accidental mortality in Cape Town (Wichmann, 2017). The low influence of coastal winds and high pollution levels are most likely responsible for the significant role of particulate matters in the prediction of COVID‐19 within Pretoria (Matyeni, 2021). The association between particulate matter and COVID‐19 cases in 17 out of 44 United States cities using the Granger causality test were found to have mean optimal lags of 7 and 13 days for PM2.5 and PM10 respectively (Kamigauti et al., 2021). In a study of 20 countries, meteorological factors have been adjudged to be good predictors of COVID‐19 cases based on the Granger causality test (Mirri et al., 2021; Sarkodie & Owusu, 2020).

## 4 Conclusion

Globally various attempts have been made to look at the influence of meteorological variables and the spread of COVID‐19 except in Africa where studies are beginning to emerge. Furthermore, studies have also highlighted the influence of the lockdown approach on the distribution of particulate matter. This study has shown conclusively that there is an improvement in air quality status as a result of the lockdown effect. This has been observed in some other studies too. The implication here is that anthropogenic activities are contributing to unhealthy air quality, especially in urban centers. Polluted air, especially in urban centers has been linked to the observed increase in COVID‐19 morbidity as a result of continued exposure by humans to these pollutants. There is therefore the need for stakeholders to ensure that standards are enforced in terms of air quality assessment.

Second, this study has shown that the influence of meteorological variables especially temperature and relative humidity are not homogeneous suggesting that other intervening variables could be responsible for the causal relationships in places where observed such as Cape Town. Other intervening variables could include the age of the populace, income status, immunity levels, lifestyle choices, diet, etc. However, this study is one of the few studies to have shown the association between meteorological factors and COVID‐19 cases in Southern Africa. From the study, temperature and relative humidity are factors that should be given special attention, especially in hinterlands. Although, there is still the need for further studies most importantly when we have longer time series to unravel the role of meteorological data in COVID‐19 transmission.

Lastly, the study has shown the significant effect of lockdown on particulate matter distribution. Even though the caveat has been pronounced, the study has shown the increasing spread of COVID‐19 across heterogeneous locations in South Africa. The intense increase in COVID‐19 cases across the second and third wave could be among other factors linked to past and present exposures to particulate matter across these localities especially in Pretoria. Therefore, there is a need for further study to investigate the continued role of meteorological parameters and COVID‐19 cases to inform comprehensive protocols that could help in the fight against these pandemic and ensure a near return to normal conditions.

## Conflict of Interest

The authors declare no conflicts of interest relevant to this study.

## Data Availability

All materials and data are publicly available and stated in the manuscript.(1) [Dataset] The particulate matter (PM1.0, 2.5, 10.0) and atmospheric parameters (temperature and humidity) were obtained from purpleair.com for Pretoria (https://www.purpleair.com/sensorlist?exclude=true&nwlat=‐25.4656400257995&selat=‐26.02173154421382&nwlng=27.601388004154018&selng=29.104648382037&sensorsActive2=604800) and Cape Town (\url{https://www.purpleair.com/sensorlist?exclude=true&nwlat=‐33.73961624522997&selat=‐34.251463795335795&nwlng=17.75585900163486&elng=19.25911937951875&sensorsActive2=604800}) (2) [Dataset] The COVID‐19 statistics were downloaded from (https://www.statista.com/statistics/1127496/largest‐cities‐in‐south‐africa/).
